# Evaluation in a Dog Model of Three Antimicrobial Glassy Coatings: Prevention of Bone Loss around Implants and Microbial Assessments

**DOI:** 10.1371/journal.pone.0140374

**Published:** 2015-10-21

**Authors:** Roberto López-Píriz, Eva Solá-Linares, Mercedes Rodriguez-Portugal, Beatriz Malpica, Idoia Díaz-Güemes, Silvia Enciso, Leticia Esteban-Tejeda, Belén Cabal, Juan José Granizo, José Serafín Moya, Ramón Torrecillas

**Affiliations:** 1 Nanomaterials and Nanotechnology Research Center (CINN-CSIC), Universidad de Oviedo (UO), Principado de Asturias, San Martín del Rey Aurelio, Asturias, Spain; 2 Advance Surgery Center Institute (ICOA), Madrid, Spain; 3 Private Practice in Dentistry, Madrid, Spain; 4 Minimally Invasive Surgery Center (CCMIJU), Cáceres, Spain; 5 School of Chemistry, Trinity College Dublin, College Green, Dublin 2, Ireland; 6 Preventive Medicine Unit, Infanta Mercedes Hospital, Parla, Spain; University of Zaragoza, SPAIN

## Abstract

**Objectives:**

The aim of the present study is to evaluate, in a ligature-induced peri-implantitis model, the efficacy of three antimicrobial glassy coatings in the prevention of biofilm formation, intrasulcular bacterial growth and the resulting peri-implant bone loss.

**Methods:**

Mandibular premolars were bilaterally extracted from five beagle dogs. Four dental implants were inserted on each hemiarch. Eight weeks after, one control zirconia abutment and three with different bactericidal coatings (G1n-Ag, ZnO35, G3) were connected. After a plaque control period, bacterial accumulation was allowed and biofilm formation on abutments was observed by Scanning Electron Microscopy (SEM). Peri-implantitis was induced by cotton ligatures. Microbial samples and peri-implant crestal bone levels of all implant sites were obtained before, during and after the breakdown period.

**Results:**

During experimental induce peri-implantitis: colony forming units counts from intrasulcular microbial samples at implants with G1n-Ag coated abutment remained close to the basal inoculum; G3 and ZnO35 coatings showed similar low counts; and anaerobic bacterias counts at control abutments exhibited a logarithmic increase by more than 2. Bone loss during passive breakdown period was no statistically significant. Additional bone loss occurred during ligature-induce breakdown: 0.71 (SD 0.48) at G3 coating, 0.57 (SD 0.36) at ZnO35 coating, 0.74 (SD 0.47) at G1n-Ag coating, and 1.29 (SD 0.45) at control abutments; and statistically significant differences (p<0.001) were found. The lowest bone loss at the end of the experiment was exhibited by implants dressing G3 coated abutments (mean 2.1; SD 0.42).

**Significance:**

Antimicrobial glassy coatings could be a useful tool to ward off, diminish or delay peri-implantitis progression.

## Introduction

Bone preservation around implants constitutes a primary criterion for implant success [1, 2]. But nowadays, one of the most important problems related to dental implant therapy is to preserve the osseointegration achieved. Peri-implantitis is an inflammatory reaction in the tissues surrounding a dental implant and is characterized by loss of supporting bone [3]. While early implant success rates are undisputed high (97–98%) [4], peri-implant disease has emerged as the main threat for implant health and depending on its severity, implant failure can occur (approximately 11% after 10 years of function) [5, 6]. In contrast to periodontal tissues, peri-implant tissues appeared to be poorly encapsulated to resolve progressive. Plaque-associated lesions are extended into the marginal bone tissue and may, if they are allowed to progress, lead to the loss of the implant [7, 8].

“Peri-implant diseases are infectious in nature” [3]. For the first time, the association between bacterial plaque biofilm formation and the pathogenesis of peri-implant diseases was demonstrated in animal studies. In this experimental model, mucositis and peri-implantitis lesions were induced by terminating the plaque control regimen and the placement and exchange of ligatures around the implant neck in a submucosal position [9].

The growing resistance of microorganisms to antibiotic therapy acquires increasingly greater concern as infectious diseases may progress uncontrolled. A particular problem is found with infections caused by biofilms, like peri-implant disease, where a variety of factors can contribute to a greater resistance to antibiotics compared with bacterias in planktonic status [10]. To cope with this problem, attention has been focused on inorganic antimicrobial solutions and great efforts have been paid in the field of nanotechnology [11]. At present, the most important research activity has been performed on materials supporting or carrier silver or copper nanoparticles [12–14]. But since the limits of systemic toxicity effects on human health of metal nanoparticles are still unknown [15], a new generation of “green antimicrobials” have been tailored to exhibit prescribed antimicrobial attributes, with microstructure and dissolution rates programmed to match each specific application and with no adverse effects on the host ecology [16, 17]. They are based on two families of glasses belonging respectively to B_2_O_3_-SiO_2_-Na_2_O-ZnO and SiO_2_-Na_2_O-Al_2_O_3_-CaO-B_2_O_3_ systems. One is a ZnO enriched glass and the other one a CaO enriched glass. The antimicrobial capability of the glass enriched with CaO relies on close contact with the target cells [18, 19]. On the contrary, in the case of the ZnO enriched glass it is attributed to the release of Zn^2+^ [20].

The aim of this study is to evaluate the efficacy of these new antimicrobial glassy coatings by inhibiting bacterial growth in subgingival sulcus and preventing peri-implant marginal crestal bone loss. For a comparative purpose, a soda-lime glass containing silver nanoparticles is also evaluated. The efficacy of this kind of glass to prevent peri-implant diseases was pointed out in previous works [21, 22]

Data from human studies are often considered to provide the highest level of scientific evidence but for ethical reasons, experimental studies of peri-implant infections cannot be conducted in humans. Hence, the information gathered in this field must rely on animal studies. Nevertheless, ethical considerations must be made also when planning experiments using animal models. In this sense Kilkenny et al. [23] presented the ARRIVE guidelines intended as a guide when preparing on animal research.

Therefore, experimental studies must be carefully designed to allow for valid outcome assessments. Since animal studies have indicated that the histopathological characteristics of these experimentally induced peri-implantitis lesions have many features in common with naturally occurring lesions [24], this model seems to have a relevance to human biology. Beagle dogs exhibit a natural susceptibility to periodontal disease [25] and reveal a jaw bone anatomy usually facilitating the insertion of common dental implants. In addition, their easy manageability facilitates postoperative oral hygiene procedures. This explains why Beagle dog appear to be the most suitable animal to conduct the ligature-induced peri-implantitis defect model.

## Material and Methods

### 2.1. Antimicrobial Glasses

In previous studies [18, 20, 26] the antimicrobial capability of different antimicrobial glasses was proven against various microorganisms. Three of those glasses were selected as precursors of the bactericidal coatings: i) a soda-lime window-like glass with silver nanoparticles labeled as G1-nAg [26], ii) a soda-lime glass from the SiO_2_-Na_2_O-Al_2_O_3_-CaO-B_2_O_3_ system labeled as G3 [18], and iii)a glass belonging to the B_2_O_3_-SiO_2_-Na_2_O-ZnO system labeled as ZnO35 [20].

Glasses without silver nanoparticles were prepared by melting appropriate mixtures of reagent grade SiO_2_, α-Al_2_O_3_, H_3_BO_3_, Na_2_CO_3_, CaCO_3_, ZnO (Sigma-Aldrich). The starting materials were weighed, mixed and melted in a Pt crucible for 1 h at 850°C to favor decarbonation of samples, and subsequently for 1 h at 1400°C (G3) and at 1250°C (ZnO35). Chemical composition of these glasses is shown in S1 Table. On the other hand, homogeneous dispersed silver nanoparticles (10–90 nm) embedded into glassy matrix with a content of silver of 20 wt.% was prepared according to the method developed by Esteban-Tejeda et al. [26].

### 2.2. Preparation of the coated abutments

Coatings were prepared following different procedures depending on the type of glass used. In the case of the glass containing silver nanoparticles, the coatings were obtained following a similar procedure that the one described in a previous work [21]. Briefly, the green coating was obtained by dipping the zirconia abutments into an ethylene glycol glass-nAg powder suspension with 70 wt.% solid content. The abutments were vertically dipped into the suspension for 3 seconds, and then withdrawn at the same speed. The resulting coatings were dried at room temperature for 24 h. The green coated abutments were subsequently heated in air atmosphere at 880°C for 2 h.

Coatings using antimicrobial glasses without silver nanoparticles were done by screen-printing technology. A polymer ink, based on a mixture of epoxy and glass powder was prepared for the glass layer screen-printing. This layer was deposited on the corresponding substrate surface. The green-coatings of ZnO35 prepared as indicated were fired in air atmosphere at 750°C for 30 min (temperature ramp 10°C/min). In the case of the green-coatings of G3 were fired in air atmosphere at 1100°C. The rate of cooling was adjusted to be rapid enough (> 300°C/min) to avoid crystallization as well any cracking or chipping phenomena.

### 2.3. Animals

The study protocol was approved by the Ethics Committee for Animal Research Welfare, Minimally Invasive Surgery Centre, Cáceres, Spain. Five male Beagle dogs were used in this experiment to accomplish with the goal of reduction, the second of the 3R’s (Replacement, Reduction and Refinement) widely accepted ethical framework for conducting scientific experiments. In addition, it is usual to employ this number of animals for this kind of experiments and satisfy statistical power requirements. Dogs at the initiation of the experiment were 12 months old and 13.5 kg mean weight.

**Fig 1:**
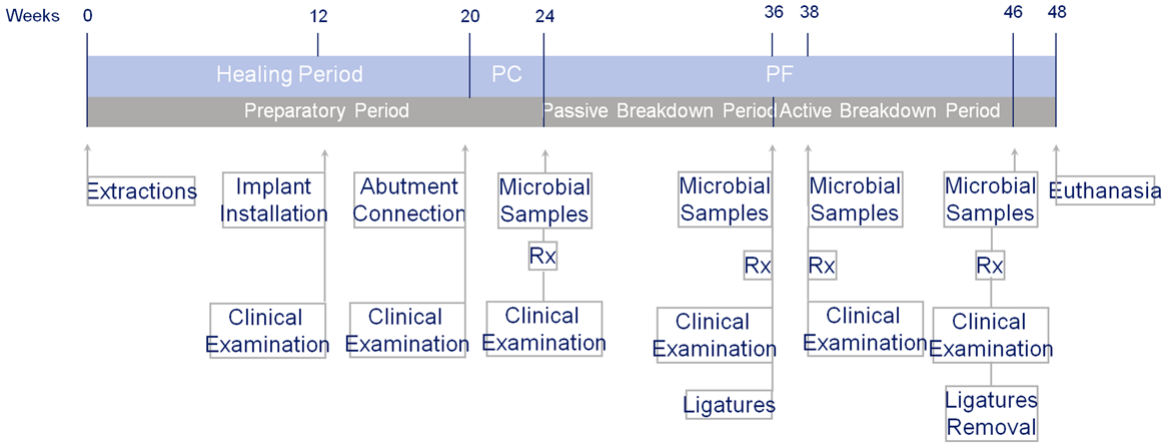
Outline of the study. This figure describes the actions conducted during the principal phases of this study. The preparatory period consisted in dental extractions, a healing period, dental implants installation, abutments connection and a plaque control regimen. During passive breakdown period all plaque control activities were stopped and afterwards, peri-implantitis was additionally induced by intrasulcular placement of cotton ligatures during an active breakdown period. PC- Plaque Control. PF- Plaque Formation.

The out line of the experiment is presented in Fig 1. Refined dog husbandry and care was provided all along the study. During all procedures, veterinary assistance was used continuously and all efforts were made to minimize suffering. General anesthesia was induced with intravenous injected propofol 10 mg/kg (Propofol Hospira, Hospira Productos Farmacéuticos y Hospitalarios, Madrid, Spain). N°7 endotracheal tube with a balloon cuff was placed and connected to a circular anesthesia circuit (Leon Plus, Heinen & Löwenstein, Bad Ems, Germany). The anesthesia was sustained with sevofluorano (Sevorane, Abbott Laboratories, Madrid, Spain). Multimodal analgesia was employed in the perioperatory (ketorolac 1 mg/kg (Toradol 30 mg, Roche);—tramadol 1.7 mg/kg (Adolonta inyec., Grünenthal); and—buprenorfine 0.01 mg/kg (Buprex, Reckitt Benckiser Pharmaceuticals Limited, Berkshire, UK).

### 2.4. Surgery

**Fig 2:**
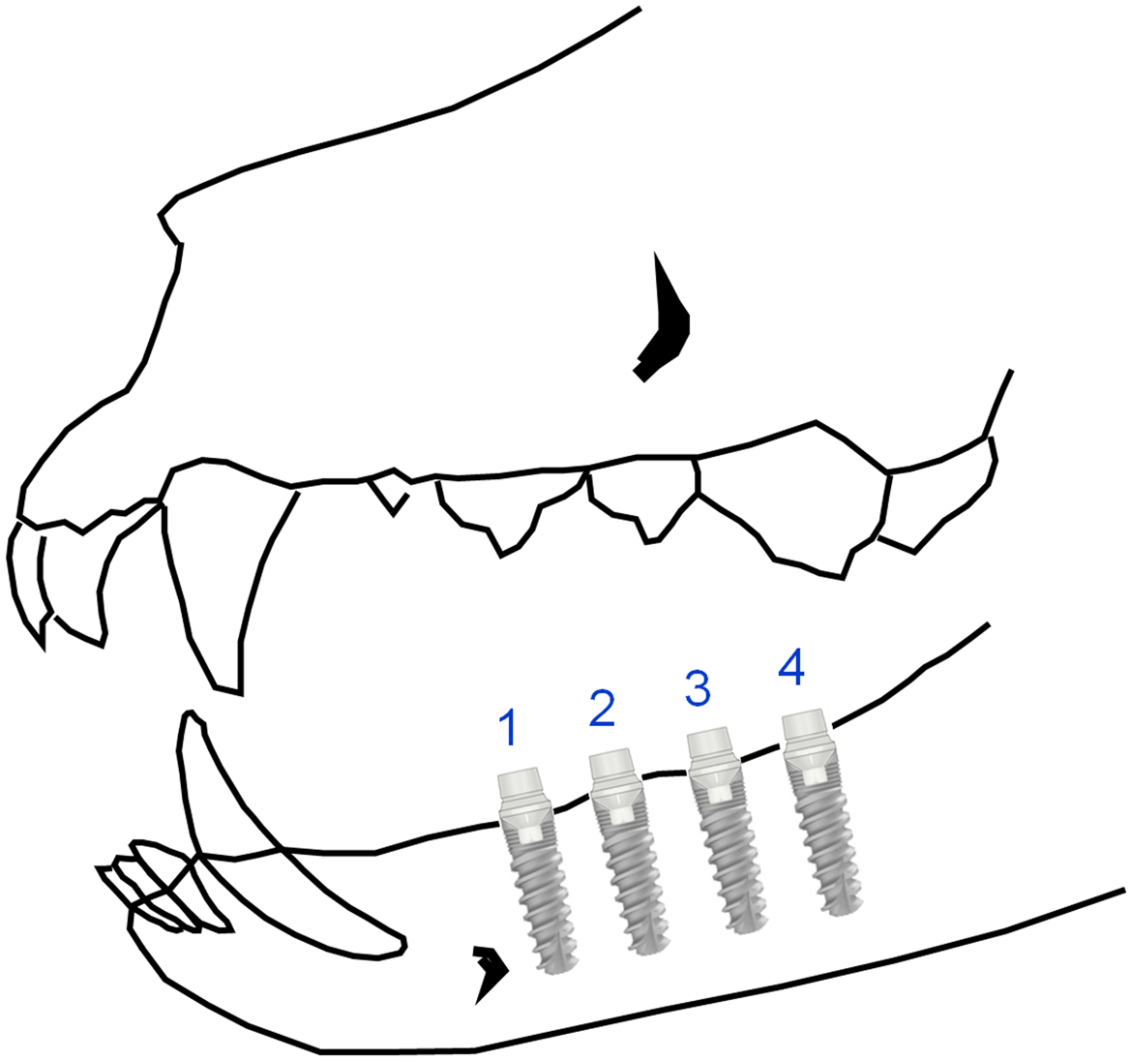
Diagram of implants locations. N°1: Control abutment. N°2: G3 coated abutment. N°3: ZnO35 coated abutmet. N°4: G1n-Ag coated abutment.

All mandibular premolars and molars were extracted. After three months of healing, mucoperiosteal flaps were elevated and 4 fixtures (MIS Implants Technologies Ltd., Israel; Implant Seven: length 10, diameter 3.75) were installed in the edentulous region on both sides of the mandible. A total of 40 implants were placed in the five male Beagle dogs. During this period animals were feed with a soft diet. Eight weeks later, second stage surgery was performed and machined zirconia abutments were attached. Mesial implants of each quadrant -implant number 1- (see Fig 2) supported machined zirconia abutments without any coating and were considered as control implants. The other 3 implants in each quadrant dressed antimicrobial glassy coated zirconia abutments, and were considered case implants. For implants in position number 2, ZnO-glassy coating was provided; for implants in position number 3, G3 glassy coating was dressed; and for implants in the most distal position n-Ag coating was allocated.

A plaque control program was initiated. This included cleaning of teeth and abutments, once a day, 5 days a week, with toothbrush and dentifrice. The plaque control regimen was terminated four weeks later. At the end of the plaque control period, animals were exposed to clinical and radiological examinations, and bacterial samples were collected. Visual signs of gingival inflammation, such as redness and swelling, were evaluated and digital radiographs were obtained from all implant sections at the beginning and the end of experiment, and twice in the transition from passive to active peri-implant breakdown period (weeks 24, 36, 38 and 46) (See Fig 1). We used a holder that allowed easy and predictable alignment of the X-ray tube, and reproducible radiographic images from which highly repeatable measurements could be made. The radiographs were analyzed using Nemotec Dental Studio^®^ Software (Nemotec SL, Madrid, Spain) and the distance between the abutment-fixture junction (A/F) and the marginal position of bone-to-implant contact (BIC) was determined. The measurements were made at both the mesial and the distal aspect of each implant and were redone after 1 week to confirm intraobserver reliability.

Bacterial samples were collected at 4 aspects of each implant (buccal, lingual, mesial and distal) by introducing sterilized paper points (n° 30, Mayfeller) into the peri-implant sulcus.

Colony forming units per milliliter (CFU/mL) were used as a measure of the number of microorganisms present in a sample. Samples were shaked and then spread uniformly on a surface of a blood-agar plate for aerobes count, on a surface of Brucella for total bacterial count, and then incubated at 35°C for 48 hours.

### 2.5. Passive breakdown period (spontaneous bacterial accumulation)

The plaque control regimen was finished and thus the plaque was allowed to accumulate during the course of the following twelve weeks. Once a week a clinical examination was performed to asses the plaque, soft tissue inflammation. At the end of this period, radiographs were obtained to assess changes in bone loss related to spontaneous plaque accumulation. Bacterial samples were also collected at this point to report CFU counts at different coatings. Afterwards, all zirconia abutments were replaced to assess coating wearing down and to ensure coating integrity during next phase of the experiment. Retrieved abutments were characterized at this point as following: The surface and cross section morphology were analyzed by Field Emission Scanning Electron Microscopy (FESEM) (FEI: Quanta FEG650) with an associated energy dispersive spectroscopy analysis (EDS) (EDAX-AMETEK).

### 2.6. Active breakdown period (ligature-induced peri-implantitis)

Cotton ligatures were placed in a submarginal position around the neck of the new abutments according to the technique described by Ericsson et al. [27] and Lindhe et al. [9]. Once a week a clinical examination was performed to asses the plaque, soft tissue inflammation and the presence of ligature. The ligatures were replaced every three weeks with new ligatures placed in the pocket of the receded gingival margin. At the end of the experimental peri-implantitis a new radiographic and clinical examination was performed. Animals were euthanized with a lethal dose of Sodium-Penthotal^®^ (B. Braun Medical SA. Rubí. Barcelona. Spain) and mandibular blocks containing fixtures were retrieved and stored in a 5% formaldehyde solution (pH 7). The implants were individual retrieved from the jaw bone using an oscillating autopsy saw.The retrieved specimens were immediately immersed in a solution of 4% formaldehyde and 1% calcium. The specimens were embedded in methyl-methacrylate and stained with combined basic fuchsin and toluidine blue. Transverse sections perpendicular to the beagle bone jaw with a thickness of approximately 80 μm were obtained for descriptive histology.

The biopsy specimens were processed immediately to obtain undecalcified thin ground sections, following Donath's method. The preparations were dyed with Harris Hematoxiline (Papanicolau, Merck, Germany) and Wheatley's modification of thrichromic stain (Chromotrope 2R, Newcommersupply, USA) and preserved with Canada balsam solution (Fluka Biochemika, USA). Preparations were examined by using a transmitted light microscope (Optiphot, Nikon, Japan) equipped with a digital Camera (DP-12, Olympus, Japan).

### 2.7. Statistical analysis

Mean values for all variables were calculated. Comparisons were made using absolute values (initial and final bone loss) and changes in a relative scale ((initial-final) / initial). Differences were analyzed in pairs (control group versus tested coated groups) using non-parametric (Mann-Whitney; Wilcoxon) methods. The null hypothesis was rejected at p<0.05.

## Results

### Characterization of the coatings before being implanted in dogs

**Fig 3:**
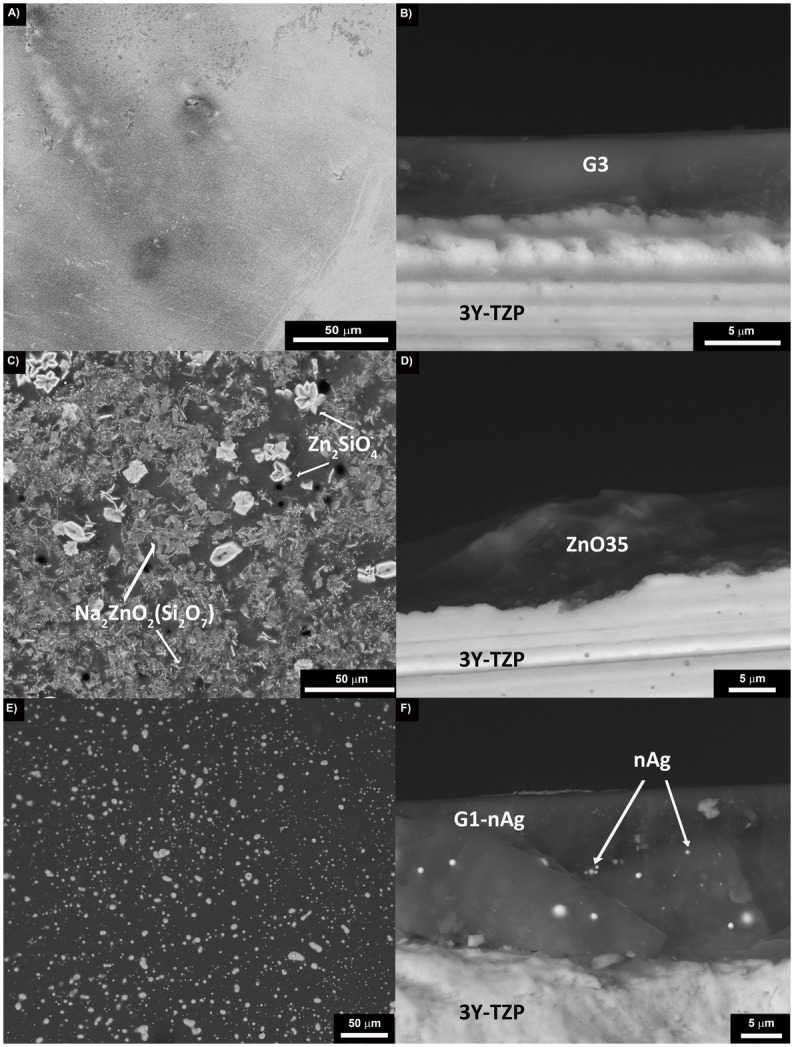
Scanning electron micrographs showing the surface of the glassy coatings: A) G3, C) ZnO35, E) G1n-Ag, and the cross section: B) G3, D) ZnO35 and F) G1n-Ag.

Scanning electron micrographs of the top surface and of the cross section of the different coatings are shown in Fig 3. No crystallization was observed in the case of the coating made of glass G3 (Fig 3A), whereas it is clearly visible when glass ZnO35 is used (Fig 3C). These crystallizations were identified by XRD (data not shown) as sodium zinc silicate crystals Na_2_ZnO_2_(Si_2_O_7_) and willemite crystals (Zn2SiO4). Thermal expansion coefficients of the glasses [α_ZnO35_ = 10.7 10^−6^ K^-1^, α_G3_ = 14.2·10^−6^ K^-1^, α_Glass-nAg_ = 11·10^−6^ K^-1^], and of the 3Y-TZP substrate (α_3Y-TZP_ = 10.6 10^-6^K^-1^) are quite similar [17], so not visible cracking was observed in any of the coatings. In the case of the coating made of glass containing silver nanoparticles (Fig 3E), silver particle size ranges between 20–90 nm but also some agglomerates (0.5–1.8 μm) are present. The average starting coating thickness was found to be 23.1 μm for G1-nAg coating, 3.6 μm for G3 coating, and 7.9 μm for ZnO35 respectively.

### Characterization of the coatings after three months being implanted in dogs

**Fig 4:**
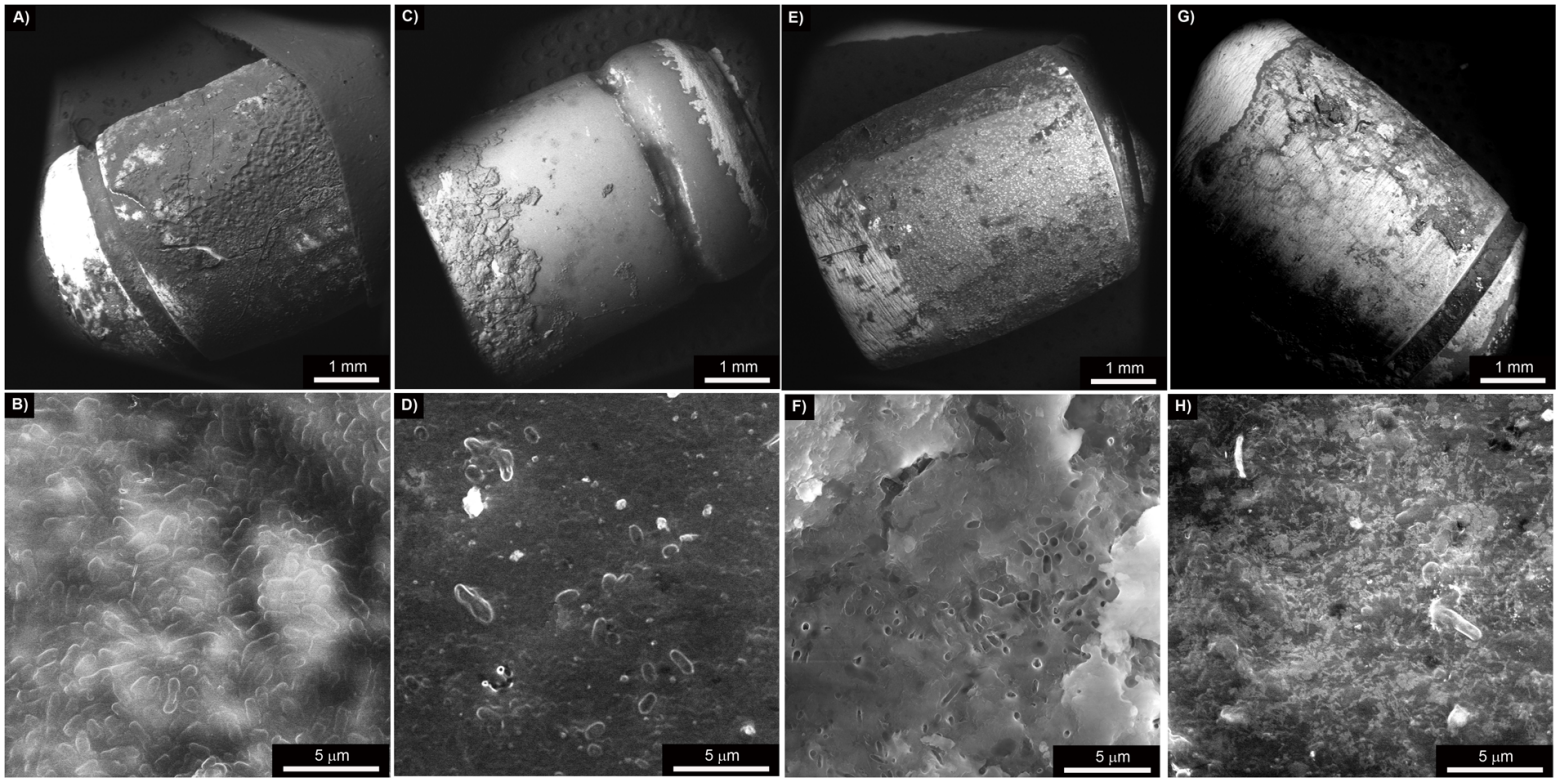
Scanning electron micrographs at different magnifications of: A and B) uncoated zirconia abutment, C and D) G3 glassy coated zirconia abutment, E and F) ZnO35 coated zirconia abutment, G and H) G1n-Ag coated zirconia abutment.

Characterization of the coatings after three months was done by scanning electron microscopy (SEM). It is worth to point out that all abutments still had part of the original coatings, although at lingual aspect G1n-Ag coating wore down significantly because of the abrasiveness of the dog’s tongue. Bacterial cells colonized densely onto the surface of the uncoated zirconia abutment (Fig 4A and 4B). Conversely, the extend of bacterial adhesion on the glass coated abutments decreased significantly, and only few microorganisms appear located on the coating. By the way of example, biofilm formation occurred only in the part of the abutment free of coating (Fig 4C) and significantly few microorganisms are shown in the abutment coated with glass G3 (Fig 4D). Biofilm formation onto the surface of ZnO35 (Fig 4E and 4F) and G1n-Ag (Fig 4G and 4H) coatings was much lower than in the case of control uncoated zirconia abutments. These micrographs are very consistent with the microbiological tests.

### Microbiological Tests

**Fig 5:**
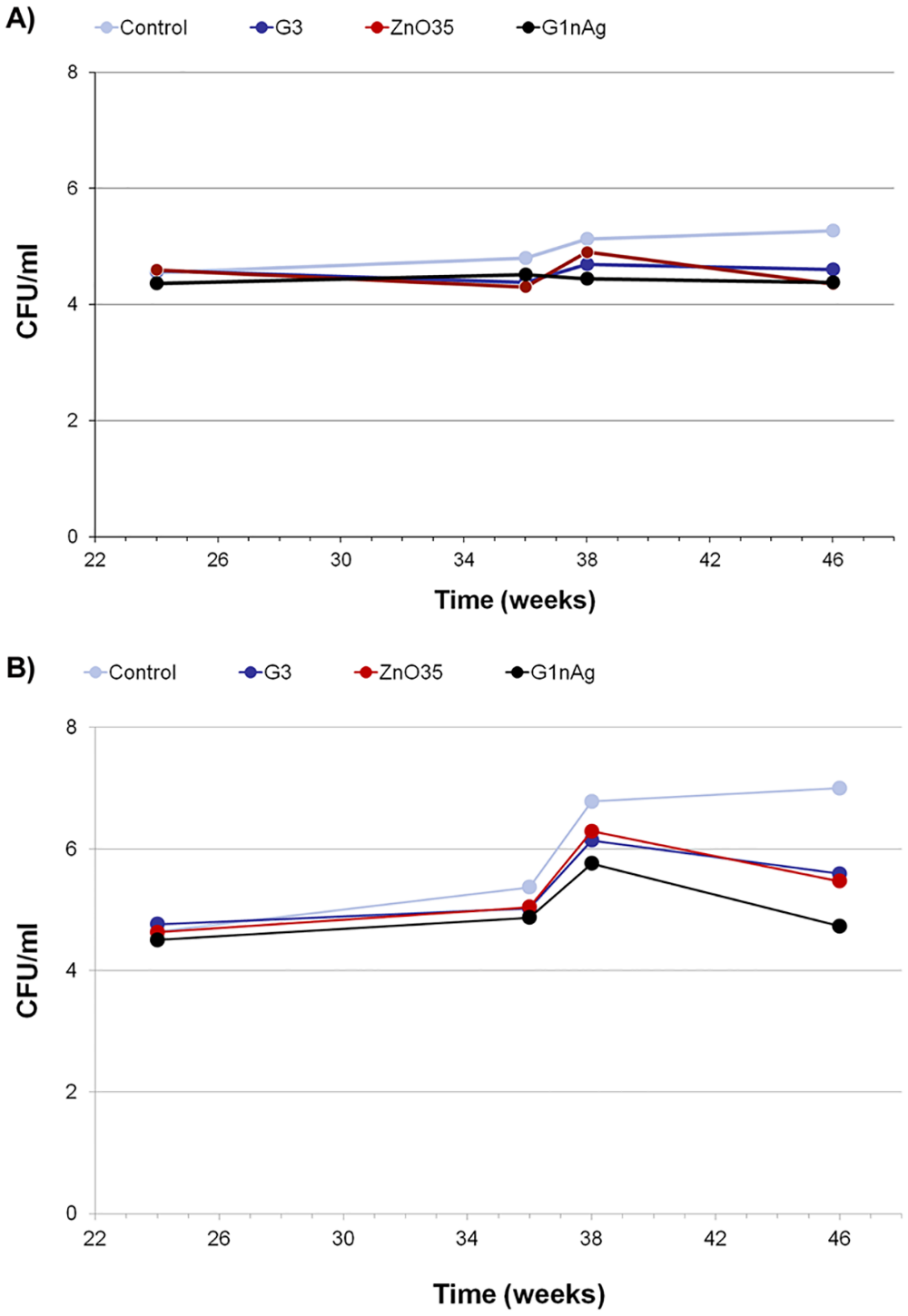
Logarithmic progression in CFU/mL of intrasulcular samples during experimental induced peri-implant disease: A) Aerobic bacterial counts, B) Anaerobic bacterial counts.

Weighted means of the CFU counts per mL are shown in Fig 5. G1n-Ag abutments maintained a baseline of anaerobic bacteria counts all along the experiment. We also emphasize that G3 and ZnO35 coated abutments had a similar effect. Conversely, control abutments without any bactericidal coating experienced a logarithmic increase of anaerobic CFU up to 2.36, which is not compatible with infection control. All coatings (G3, ZnO35 and G1nAg) developed along similar lines for aerobic bacteria, with CFU means closed to the primary inoculum. On the contrary, control abutment exhibited an increase over time.

### Clinical examinations

**Fig 6:**
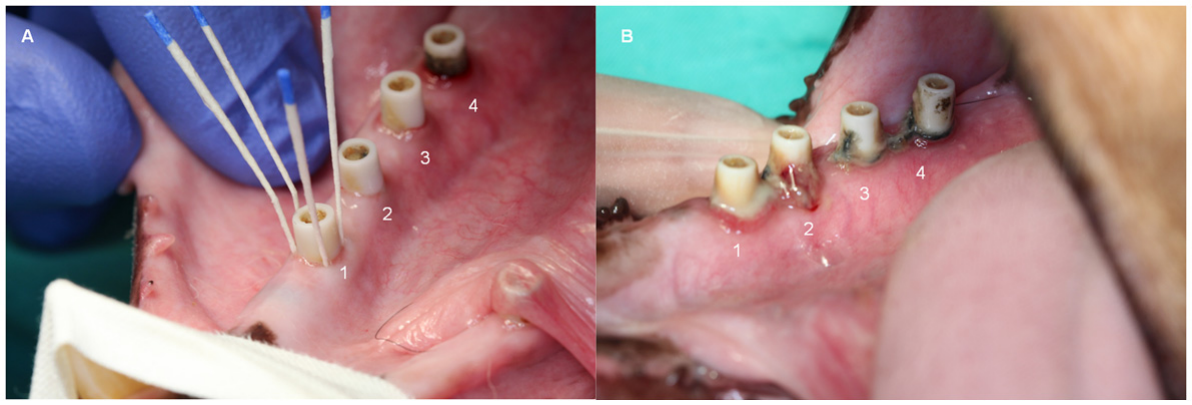
A) Intrasulcular sampling with sterile paper points at week 24. B) Clinical view of gin- gival inflammation at week 46. 1. Uncoated zirconia abutment, 2. G3 glassy coated abutment, 3. ZnO35 coated abutment, 4. G1n-Ag coated abutment.

At the second stage surgery, one implant planned to be used as a G1n-Ag case-implant was lost (dog 794 microchip code). Therefore a total of 29 implants were successfully osseointegrated. Clinical examinations performed at the beginning of the experimental induced peri-implantitis period revealed important gingival inflammation around the G1n-Ag and slight inflammation around ZnO35 biocidal coated abutments and minimal changes around control implant’s gingiva. These observations were progressively changing over, so that at the end of the experiment control implants presented important gingival inflammation while all biocide coated implants showed minimal gingival changes (Fig 6). A large amount of plaque was harbored at the control implants while at the biocidal coated implants plaque retention was located at ligature, despite the rough surface of the coating. There were no adverse events during experimental procedures.

### Radiographic assessments

**Fig 7:**
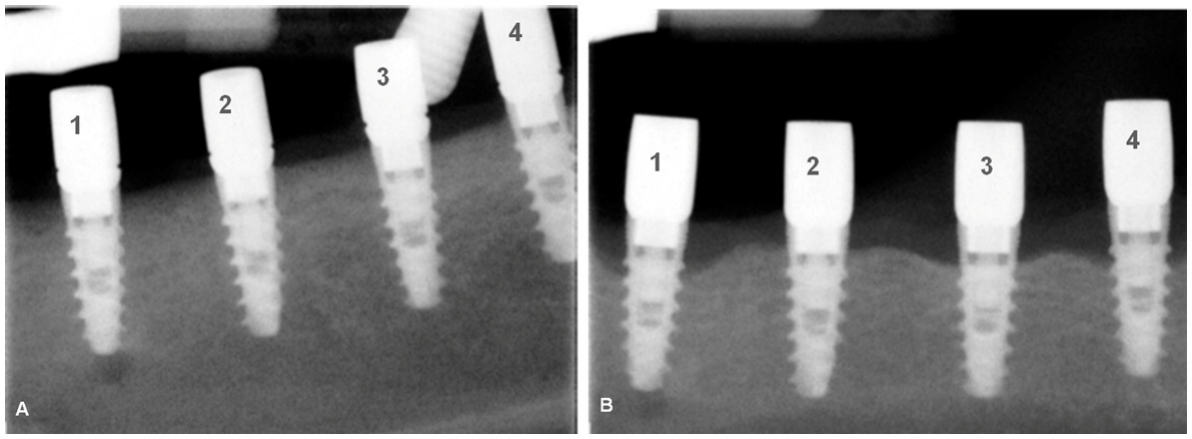
Digital radiographs of implants at the beginning of the plaque formation period in week 24 (A) and at the end of the study in week 46 (B). Differences in crestal bone level are evident. 1. Uncoated zirconia abutment, 2. G3 glassy coated abutment, 3. ZnO35 coated abutment, 4. G1n-Ag coated abutment.

**Fig 8:**
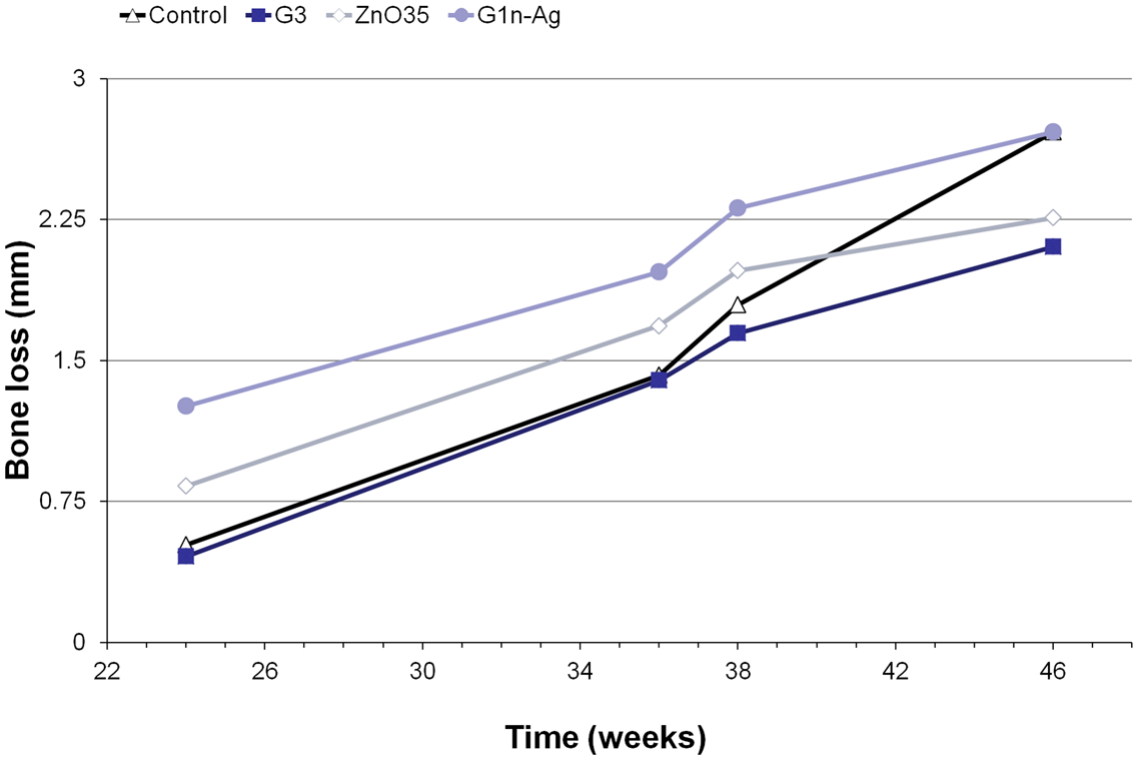
Bone loss progression at different coatings during experimental induced peri-implantitis period.

The distance from the implant shoulder to the alveolar bone crest changed to a greater or lesser extend depending on the abutment coating (Fig 7). Fig 8 displays bone loss progression at different coatings during experimental induce peri-implantitis (passive breakdown -weeks 24 to 36- plus active breakdown -weeks 36 to 46-); and S2 and S3 Tables show statistical results.

From Fig 8 we can see that at the end of plaque control period (week 24) bone loss mean values were 0.5mm (SD 0.44) at position 1; 0.45mm (SD 0.39) at position 2; 0.8mm (SD 0.77) at position 3; and 1.2mm (SD 0.47) at position 4. Significant statistical differences (p<0.001) in bone loss were found between position 4 and positions 1 and 2, but this was no the case for position 1 vs 2 (p 0.86), 1 vs 3 (p 0.31), 2 vs 3 (p 0.16) and 3 vs 4 (p 0.026).

Once this initial and spontaneous bone remodeling is originated and plaque accumulation is allowed, no statistical significant (p 0.29) differences in mean bone loss around implants are showed during passive breakdown period (S2 Table). On the contrary, during the active breakdown period (ligature induced peri-implantitis) additional bone loss occurred (Fig 8). This additional bone loss varied considerably between control and case implants (S2 Table). In an absolute scale, the difference variable (initial mean value—final mean value) shows a significant additional bone loss at control implants (p<0.001). When we used a relative scale ((initial-final)/ initial) to quantify additional bone loss during active breakdown period a percentage change of 221% was observed at control implants. Percentages of additional bone loss observed in implants dressed with a biocidal coated abutment were about 124% lower (p<0.001) than the control ones.

Total bone loss expressed in absolute values was 2.21 mm mean (SD 0.46) for control abutment, 1.64 (SD 0.43) for G3 coating, 1.42 (SD 0.40) for ZnO35 coating, and 1.45 (SD 0.56) for G1n-Ag coating. The lowest final mean bone loss was exhibited by implants that dressed G3 coated abutments (mean 2.1; SD 0.42). From S3 Table we can infer that significant statistical differences (p<0.001) in absolute (difference) and relative (change) mean bone loss values were found between coated abutments and control ones.

The results from the reproducibility assessments of the radiographic measurements revealed small differences between the two assessments. The intra-observer mean difference was 0.09, and variance and standard deviation were 0.06 and 0.23 respectively.

**Fig 9:**
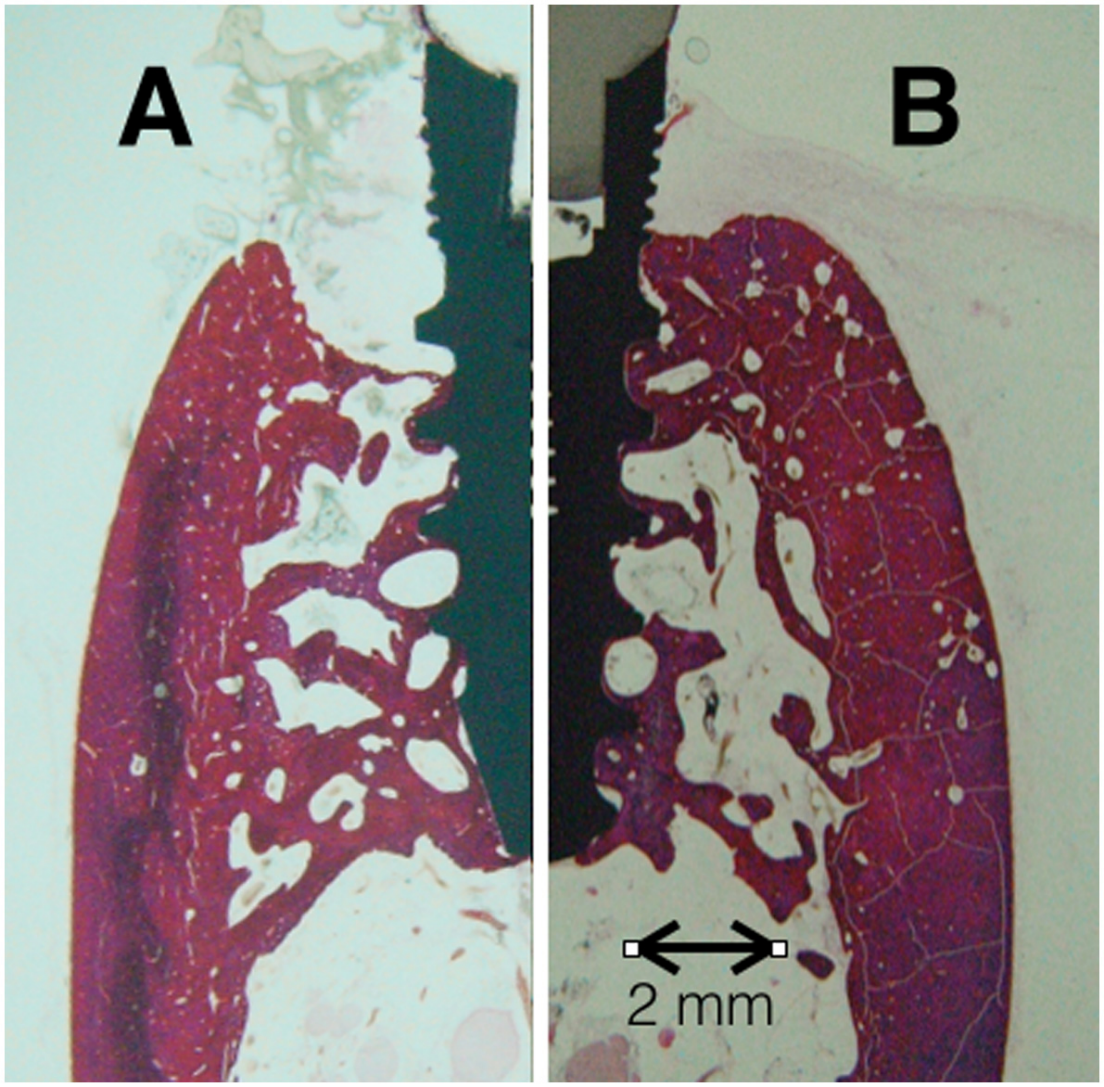
Transversal sections of implants at the end of the study in week 48. After a passive plaque formation period and an active ligature induce peri-implantitis period: A) An implant wearing uncoated zirconia abutment shows a bone resorption process that forms a crater defect and reduces bone-to-implant contact level to the macro-threads zone of the implant. B) An implant wearing G3 glassy coated zirconia abutment undergoes an evident lesser bone loss limited to the micro-threads zone and does not display the typical crater shape bone defect associated with active peri-implantitis.

All of the above is in accordance with results presented in Fig 9, where cross section A (corresponding with an implant wearing uncoated zirconia abutment) shows a bone resorption process that forms a crater defect and reduce bone-to-implant contact level to the macro-threads zone of the implant; and cross section B (corresponding with an implant wearing G3 glassy coated zirconia abutment) sustain an evident lesser bone loss limited to the micro-threads zone and do not incur in the typical crater shape bone defect associated with active peri-implantitis. A completed histological and histomorphometric analysis of the different gross sections obtained from the implants is in progress.

## Discussion

In the present study microbial and marginal bone levels at implants subjected to experimental peri-implant disease were analyzed. The different types of abutments included in the experiment, which differed with regards to coating, exhibited dissimilar competence for effective microbial load control and bone resorption around implants.

Scanning electron microscopy (SEM) of the coatings three months after being implanted in dogs proved that structural integrity is preserved. It is further observed that glassy coatings decrease bacterial attachment and proliferation on its surface (Fig 4). Large clusters of bacteria can be clearly observed on control abutment, while only few bacillus shaped bacteria are barely spotted. In the same line other studies showed inorganic nanoparticles as a coating material to inhibit bacterial adhesion and promote osteoblast growth [28].

CFU counts from intrasulcular samples resulted in consistent findings of SEM abutment analysis. No statistical significant differences were detected in aerobic bacterial counts between control or coated abutments, but a slight increase in CFU from basal inoculum was evidence in control abutments as experiment evolved. It should be noted that, whilst control abutments experimented a logarithm of increase > 2 for anaerobic cfu/ml, the antimicrobial study of these glassy coatings pinpointed that this new family of bactericidal glasses is very effective maintaining anaerobic bacterias levels close to the basal inoculum logarithmic concentration (Fig 5B).

“In vitro” experiments previously reported [17, 29, 30] had documented that this new family of biomaterials is very effective diminishing (logarithm of reduction >3) the growth of bacteria (such as *S*. *aureus*, *E*. *coli*, *S*. *epidermidis*, *M*. *lutea* and *P*. *aeruginosa*) as well as yeast (*C*. *Krusei*). Microbiological results regarded from this dog experiment allow us to assert that the same “in vivo” microbial counts control effectiveness is exhibited. This is also quite consistent with clinical findings (Fig 4), where at week 24 minimal gingival inflammation is observed at positions 3 (ZnO35) and 4 (G1n-Ag) and no gingival changes at all can be pinpointed at position 1 (control) and 2 (G3). Nevertheless, at the end of induce peri-implant disease the reverse happens. At week 46 plenty of suppuration arise from sulcus in control abutment position 1 and adversely affecting the medial aspect of position 2 (G3); it is noteworthy that positions 3 (ZnO35) and 4 (G1n-Ag) hardly exhibit any gingival inflammation in spite of bacterial accumulation caused by sub-gingival cotton ligatures.

Previous experiments [9, 31, 32] have demonstrated that mucositis and peri-implantitis may be induced by terminating the plaque control regimen (passive breakdown) and the placement and exchange of ligatures around the implant neck in a sub-mucosal position (active breakdown). Fig 7 shows radiological crestal bone level at the beginning and end of peri-implant induce disease. All implants exhibited bone loss to a greater or lesser extend depending on the abutment coating dressed.

The mean amount of bone loss that occurred during the preparatory period (implant insertion-plaque control cessation) can be seen from Fig 8. Mesial and distal aspect of each implant were considered observation units (n = 20) to assess bone loss. All abutments with the exception of G1n-Ag, experimented similar bone loss during plaque control period that could possibly be explained by biological width development. Similar results occurred in an earlier study looking at bone loss around implants wearing G1n-Ag coated abutments [21]. This spontaneous bone loss around implants dressed with G1n-Ag coated abutments during plaque control phase, and previous to the induce peri-implant disease period, could be related to a time-limited minimal gingival inflammatory reaction triggered by the coating material itself (Fig 6). Similar inflammatory reaction was shown to a lesser extend by ZnO35 coated abutments, but no inflammatory changes were triggered by control nor G3 coated abutments.

No statistical differences (p<0.29) in bone loss have been shown during passive breakdown period. This is in concordance with the slight increase of bacterial counts assessments from intrasulcular samples at this period. It is worth noting that during ligature induce peri-implantitis (active breakdown period) differences in bone loss were statistically significant (p<0.001) between control abutment and all coated abutments, both for absolutes values (difference variable) and for relative values (change variable). This is directly linked to anaerobic bacterial logarithmic increase caused by ligatures. The more bacterial counts increase the more bone loss exhibited around implants. G1n-Ag showed the best bacterial control counts (Fig 1B) and one of the lowest bone loss in absolute values (total difference) (S2 Table). Nevertheless, due to the time-limited spontaneous bone resorption that these coated abutments exhibited during plaque control period, from Fig 8 we can see that total final bone loss at week 46 was similar to control abutments. Antimicrobial properties of ZnO35 and G3 coatings were similar and closed to G1n-Ag coating, and therefore bone loss during ligature induce peri-implantitis around implants that dressed these coatings (positions 2 and 3) resulted statistically significant (p<0.001) low when compared with control abutments. Indeed, from S2 Table can be seen than ZnO35 coating exhibited the lowest total bone loss expressed in an absolute value (initial bone level assessment minus final bone level value). However, as in the case of G1n-Ag coating, ZnO35 coatings went through a time-limited bone resorption process during initial plaque control period, although to less extension. This provoked a total final bone loss of 2.25 mm at week 46 even though it has exhibited exceptional antimicrobial properties during active breakdown period. It is noteworthy that G3 coated abutments exhibited the lowest mean bone loss compared with controls or any other antimicrobial coating all along the experiment and at the final point at week 46.

It is realized that there is a concern with metal-based nanomaterials—engineered metal and metal oxide nanoparticles—to human health [33], but in spite of all efforts made to clarify the limits of such toxic health effects, there is still insufficient evidence to support the claim [15, 34].

In the present study animals did not exhibit any coating related adverse effect further than initial time-limited gingival inflammation assessed around ZnO35 and G1n-Ag coatings. It should be pointed out that G3 coating did not exhibit this initial gingival inflammation. Any health hazard risk was showed neither in clinical nor analytical evaluations of animals performed all along the experiment.

Experimental models in animals were established to evaluate tissue reactions in relation to the use of dental implants. It is realized that this model may not mimic the onset and/or progression of natural disease in humans, but comparisons between lesions produced with such models exhibit features similar to those found in human biopsy material [24]. It is acknowledged that ethical considerations prevent complete analysis of pathogenesis on peri-implant diseases in humans, which explains the use of preclinical *in vivo* models.

## Conclusions

As it is evident from the results presented in this study, all the studied glassy coatings exhibit antimicrobial properties that avoid bacterial attachment to abutment and keep bacterial proliferation under control in the peri-implant sulcus. These glass coatings are particularly effective preventing anaerobic bacterial growth, which are the ones directly associated with the peri-implant disease.

Further, our analysis indicates that radiologic resorption around implants due to experimentally induced peri-implantitis is significantly large in implants dressed with control abutments compared with implants dressed with glassy antimicrobial coatings. This bone resorption prevention is especially apparent in G3 coated abutments and also in less extension in the case of ZnO35 coated abutments.

There is a need for an analysis of the clinical course of bone resorption around coated implants placed in patients with high risk of peri-implantitis. Therefore, clinical trial designs dealing with antimicrobial glassy coatings are an urgent must. In clinical practice, it shall be develop and implement suitably effective coating strategies on definitive prosthetic transgingival abutments or on zirconia crowns.

## Supporting Information

S1 Table Chemical compositions of the glasses (mol%). (DOC)

S2 Table Statistical results of bone loss. ***Abutment:** 1: Control abutment. 2: G3 abutment. 3: ZnO35 abutment. 4: G1-nAg abutment*.
***Difference:** Initial bone level assessment minus final value. (Eg: Difference weeks 24–36 = bone level in week 24 –bone level in week 36)*.
***Change:** Initial bone level—final bone level/initial bone level. Change represent a porcentual value in bone level loss*.
***Statistical significance:** Global significance including all groups (Kruskal-Wallis test)*.
(DOCX)

S3 Table Statistical significance in bone loss difference and change during Active Breakdown Period (weeks 36–46). ***Abutment:** 1: Control abutment. 2: G3 abutment. 3: ZnO35 abutment. 4: G1-nAg abutment*.
***Difference:** Initial bone level assessment minus final value. (Eg: Difference weeks 24–36 = bone level in week 24 –bone level in week 36)*.
***Change:** Initial bone level—final bone level/initial bone level. Change represents a porcentual value in bone level loss*.
***Statistical significance:** Paired significance between groups (Mann Whitney test)*.
(DOCX)
