# Supplementary material for: Evaluation in a Dog Model of Three Antimicrobial Glassy Coatings: Prevention of Bone Loss around Implants and Microbial Assessments
Source: PLoS One. 2015 Oct 21;10(10):e0140374. doi: 10.1371/journal.pone.0140374 (PMC4619200; doi:10.1371/journal.pone.0140374)
Supplement: S1 Table — (DOC) [file pone.0140374.s001.doc]

|  | **SiO2** | **B2O3** | **Na2O** | **CaO** | **Al2O3** | **K2O** | **ZnO** |
| --- | --- | --- | --- | --- | --- | --- | --- |
| **ZnO35** | 23.1 | 35.3 | 6.4 | - | 3.6 | - | 31.6 |
| **G3** | 43.0 | 7.8 | 19.4 | 22.0 | 7.4 | 0.4 | - |

Table 1. *Chemical compositions of the glasses (mol%).*
