# Supplementary figures and images for: Evaluation in a Dog Model of Three Antimicrobial Glassy Coatings: Prevention of Bone Loss around Implants and Microbial Assessments
Source: PLoS One. 2015 Oct 21;10(10):e0140374. doi: 10.1371/journal.pone.0140374 (PMC4619200; doi:10.1371/journal.pone.0140374)

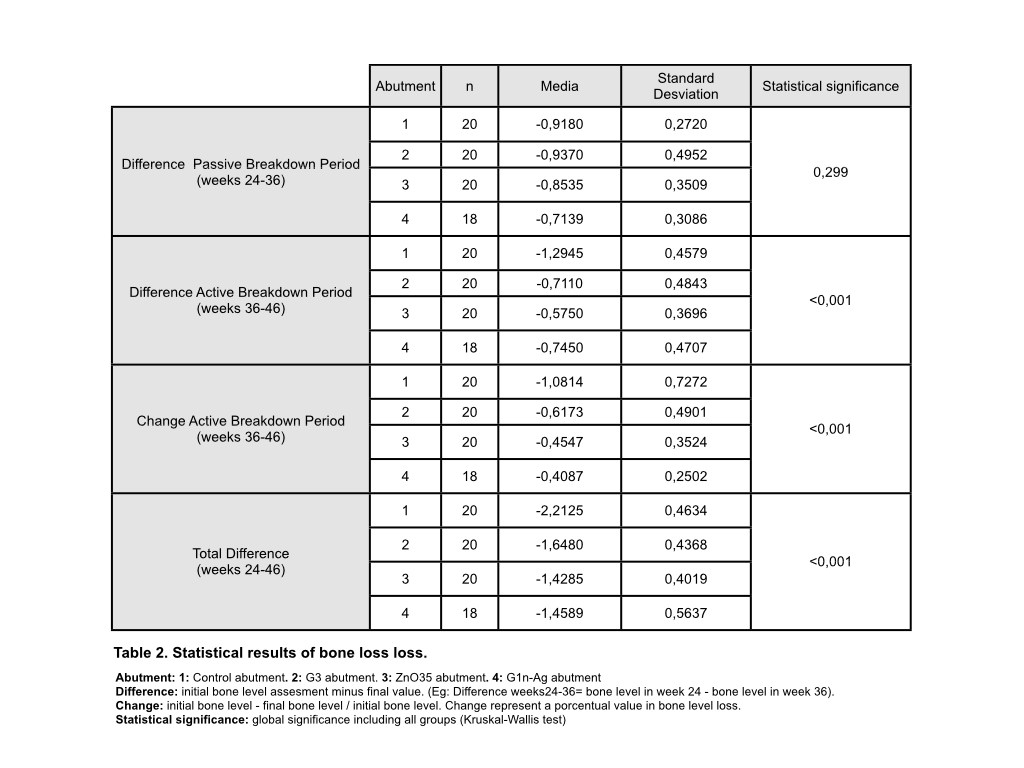

Supplement: S2 Table — Abutment: 1: Control abutment. 2: G3 abutment. 3: ZnO35 abutment. 4: G1-nAg abutment. Difference: Initial bone level assessment minus final value. (Eg: Difference weeks 24–36 = bone level in week 24 –bone level in week 36). Change: Initial bone level—final bone level/initial bone level. Change represent a porcentual value in bone level loss. Statistical significance: Global significance including all groups (Kruskal-Wallis test). (DOCX) [file pone.0140374.s002.docx]

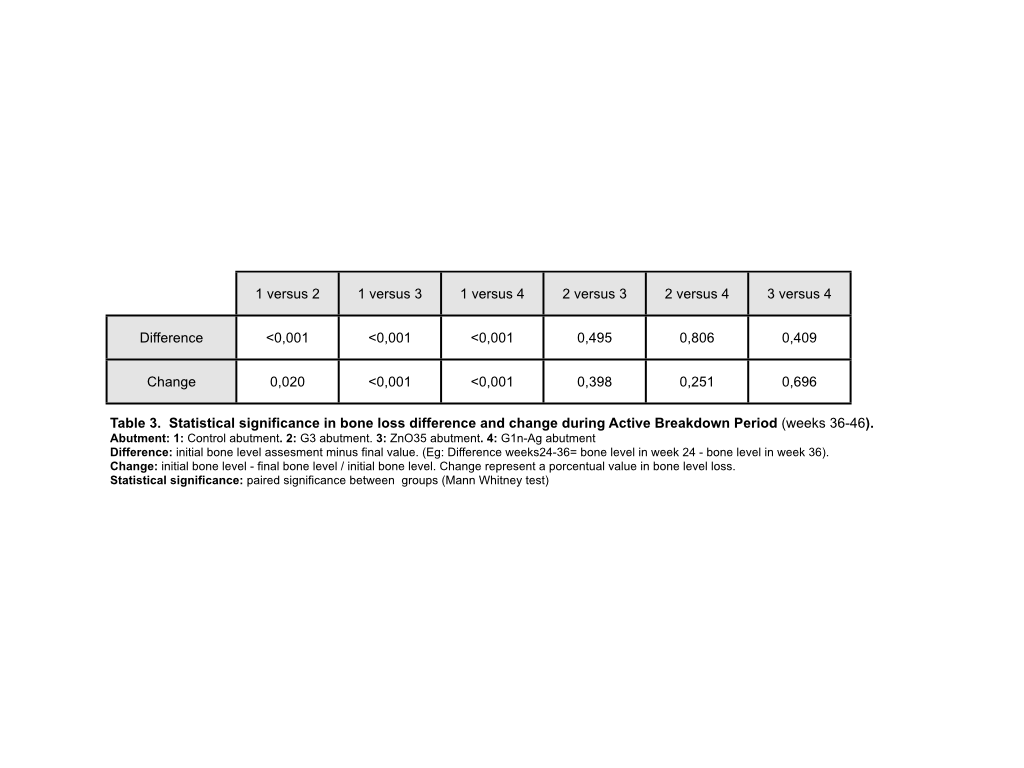

Supplement: S3 Table — Abutment: 1: Control abutment. 2: G3 abutment. 3: ZnO35 abutment. 4: G1-nAg abutment. Difference: Initial bone level assessment minus final value. (Eg: Difference weeks 24–36 = bone level in week 24 –bone level in week 36). Change: Initial bone level—final bone level/initial bone level. Change represents a porcentual value in bone level loss. Statistical significance: Paired significance between groups (Mann Whitney test). (DOCX) [file pone.0140374.s003.docx]
